# Western Iowa Veterinary Medical Association

**Published:** 1896-01

**Authors:** P. F. Gaunt

**Affiliations:** Secretary; Per G. A. J.


Secretary.

### WESTERN IOWA VETERINARY MEDICAL ASSOCIATION.

This Association met, pursuant to a call of the Secretary, in the room of the Sioux City Scientific Association, on November 26, 1895, with President Millar in the chair. Members present: Drs. H. Shipley, G. E. Armstrong,

C. J. Hinkley, P. F. Gaunt, J. J. Millar, and G. A. Johnson, with G. J. Ross, M.D., and C. R. Mock as visitors. During the routine of business Dr. Johnson presented a draft of a bill for legislation controlling milk and meat inspection in the State. After a discussion, entered into by all the members present, the Secretary was instructed to draft a set of resolutions that it is the sense of this Association that such a bill should become a law, and forward the same to the Legislature when it shall have met in Des Moines.

Dr. Johnson then presented a paper on "The Technique of the Tuberculin Test,"[1] which was discussed by all present. Dr. Gaunt presented a report of an outbreak of rabies, which elicited a full discussion by all present. The meeting adjourned to meet at the call of the Secretary.

P. F. GAUNT, Secretary.
Per G. A. J.

### CONNECTICUT VETERINARY MEDICAL ASSOCIATION.

[1] Will appear in February number.

